# Supplementary material for: High Non-Cardiac Death Incidence Should Be a Limitation of Drug-Eluting Stents Implantation? Insights from Recent Randomized Data
Source: Diagnostics (Basel). 2023 Apr 2;13(7):1321. doi: 10.3390/diagnostics13071321 (PMC10093159; doi:10.3390/diagnostics13071321)
Supplement: Supplementary file 1 [file diagnostics-13-01321-s001.zip › diagnostics-2228268-supplementary.pdf]

Table S1: Randomized studies reporting high incidence of non-cardiac death or cancer.

	Design	Comparator	Patients			Reference
BIOSCIENCE	RCT	DES3 vs DES2	2119	Target lesion failure	5 years	[3]
ISCHEMIA	RCT	Invasive (74% DES/26% CABG) vs OMT	5179	Composite of death from cardiovascular causes, myocardial infarction, or hospitalization for unstable angina, heart failure, or resuscitated cardiac arrest	3.2 years	[5]
ISCHEMIA EXTENDED	RCT	Invasive (74% DES2/26% CABG) vs OMT	4825	Death, cardiovascular death, non-cardiac death	5.7 years	[7]
REVIVED	RCT	DES2/3 vs OMT	700	Death from any cause or hospitalization for heart failure	3.4 years	[6]
EXCEL	RCT	DES2 vs CABG	1905	Any cause of death+ stoke +myocardial infarction	5 years	[4]
Nordmann et al	Meta-analysis	DES1 vs BMS	8221	Death, cardiovascular death, non-cardiac death	4 years	[11]
Gaudino et al	Meta-analysis	BMS vs CABG<br>DES1 vs CABG<br>DES2 vs CABG	13620	Death, cardiovascular death, non-cardiac death	5 years	[16]

RCT: randomized clinical trials, DES3: third generation drug eluting stent, DES2: second generation drug eluting stent, CABG: coronary artery bypass surgery, OMT: optimal medical treatment, BMS: bare metal stents, DES1: first generation drug eluting stents.
